# Working dogs cooperate among one another by generalised reciprocity

**DOI:** 10.1038/srep43867

**Published:** 2017-03-06

**Authors:** Nastassja Gfrerer, Michael Taborsky

**Affiliations:** 1 Behavioural Ecology, Institute of Ecology and Evolution, University of Bern, Wohlenstrasse 50a, CH-3032 Hinterkappelen, Switzerland

## Abstract

Cooperation by generalised reciprocity implies that individuals apply the decision rule “help anyone if helped by someone”. This mechanism has been shown to generate evolutionarily stable levels of cooperation, but as yet it is unclear how widely this cooperation mechanism is applied among animals. Dogs (*Canis familiaris*) are highly social animals with considerable cognitive potential and the ability to differentiate between individual social partners. But although dogs can solve complex problems, they may use simple rules for behavioural decisions. Here we show that dogs trained in an instrumental cooperative task to provide food to a social partner help conspecifics more often after receiving help from a dog before. Remarkably, in so doing they show no distinction between partners that had helped them before and completely unfamiliar conspecifics. Apparently, dogs use the simple decision rule characterizing generalised reciprocity, although they are probably capable of using the more complex decision rule of direct reciprocity: “help someone who has helped you”. However, generalized reciprocity involves lower information processing costs and is therefore a cheaper cooperation strategy. Our results imply that generalised reciprocity might be applied more commonly than direct reciprocity also in other mutually cooperating animals.

Reciprocity can explain cooperation among unrelated individuals, where two or more individuals help each other in turn^1^. Past interactions influence the decision to cooperate in future encounters. Nevertheless, direct reciprocity - A helps B because B has helped A before – has been assumed to occur only in animals with specific cognitive abilities^2^,^3^,^4^,^5^. Individuals must remember with whom they interacted before, and how a particular social partner behaved towards them in previous interactions^3^. Evidence for direct reciprocity among animals exists both from observations in nature and from experimental studies^6^,^7^,^8^,^9^,^10^,^11^,^12^,^13^, reviewed in refs 14, 15, 16, 17, 18. In contrast to direct reciprocity, generalised reciprocity implies that individuals help others if they received help from someone else. This is cognitively much less demanding, because individuals do not need to remember with whom they previously interacted and how certain individuals previously behaved towards them^19^,^20^,^21^. Hence, generalised reciprocity works without individual recognition and specific social memory, while it can still generate evolutionarily stable levels of cooperation under a wide range of conditions^20^,^21^,^22^,^23^,^24^,^25^. So far, generalised reciprocity has been demonstrated in humans^26^,^27^, capuchin monkeys^28^ and Norway rats^29^. As organisms lacking cognitive abilities such as bacteria and plants are known to cooperate mutually among one another, it has been suggested that simple decision rules may be sufficient for reciprocal cooperation instead of involving cognitive complexity^30^.

Domestic dogs (*Canis familiaris*) successfully perform cooperative tasks, resolve complex social conflicts and show strategic behaviour in interactions with conspecifics and humans^31^,^32^. In a recent study they were shown to donate food to a familiar conspecific in a bar pulling task^33^. Nevertheless, despite their capacity extending beyond the application of simple rules, dogs have been shown to use simple cognitive mechanisms for behavioural decisions^34^. This confirms theoretical predictions that simple ”rule of thumb” solutions to complex problems, rather than specific strategies, should underlie the behaviour of animals^20^,^21^,^22^,^23^,^25^,^35^,^36^.

Reciprocal cooperation involves costly advance payment at uncertain returns. This uncertainty can be reduced by repeatedly interacting with the same social partner, which enables tit for tat – like stable cooperation^37^,^38^. Hence, if animals make optimal decisions when given the choice to apply either direct or generalised reciprocity, they should prefer the former because iterated exchanges of goods and services with the same partner provide safer and more immediate returns^8^,^22^,^26^. Nevertheless, the costs of monitoring and remembering the behaviour of individual interaction partners may outweigh the benefits of higher predictability, which might lead to a preferential use of the simpler decision rule characterizing generalised reciprocity.

In this study, we test whether highly social animals like domestic dogs prefer using simple or complex mechanisms when deciding whether to cooperate with a social partner. Dogs are able to differentiate between conspecifics^39^ and possess considerable cognitive skills including memory of socially learned tasks^40^ which are prerequisites for applying direct reciprocity. Here we ask whether dogs reciprocate help received from a conspecific at all, and if so, whether they make use of their cognitive capacity when deciding to help a social partner in an iterated prisoner’s dilemma paradigm (IPD), or whether instead they apply the simpler and cheaper decision rule characterizing generalised reciprocity.

## Results

### Direct and generalised reciprocity tested separately

**Figure 1:**
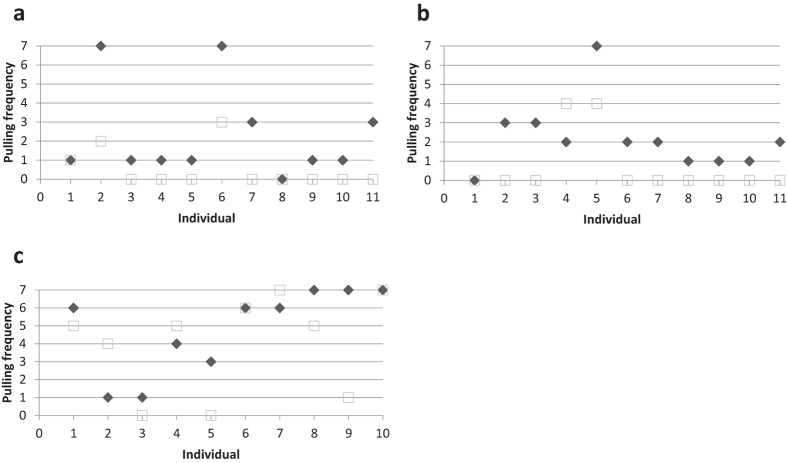
Reciprocal cooperation in dogs. (**a**) Direct reciprocity: dogs showed a higher propensity to pull for a partner that had helped them before (cooperator treatment, *black diamonds*) than for a partner that had not them helped before (non-cooperator treatment, *open squares*). (**b**) Generalised reciprocity: dogs showed a higher propensity to pull for an unknown partner after having received help from other social partners than after having received no help (*symbols as above*). (**c**) Direct vs generalised reciprocity: the pulling frequencies of dogs did not differ between situations in which they could pay back received help to the previous helper (direct reciprocity paradigm, *black diamonds*) or to a different individual (generalised reciprocity paradigm, *open squares*).

When direct and generalised reciprocity were tested separately, the dogs pulled more often after experiencing help than when not being helped before in both paradigms (paired Wilcoxon signed-ranks tests; n = 11, V = 45, p = 0.008 for direct reciprocity, Fig. 1a; n = 11, V = 49.5, p = 0.027 for generalised reciprocity, Fig. 1b). There was no difference between direct and generalised reciprocity in the cooperator treatment (generalized linear mixed model; n = 11, Df = 1, p = 0.744).

### Direct versus generalised reciprocity

**Figure 2:**
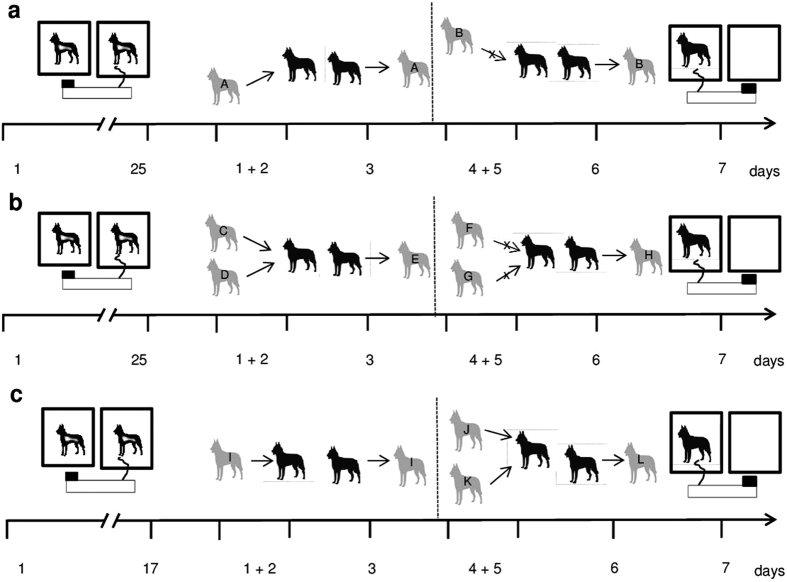
Experimental setup to test whether dogs cooperate in a reciprocal food exchange task. Focal dogs are drawn in black, social partners are depicted in grey. The time schedule (days) is shown beneath the graphs. Pre-experimental training (25 days in Fig. 1a,b; 17 days in Fig. 1c) and experimental test; two dogs were combined in adjacent kennels, but separated from each other by a 60 cm gap to prevent physical interactions. One dog produced food for the partner dog by pulling a rope attached to a mobile platform, while it was not rewarded itself for this behaviour. The graphs show a particular example, but the order of treatment presentations was randomized. Two days of experience (days 1 + 2/days 4 + 5) were followed by a test day (day 3/day 6), where the focal dog (black) was tested in the role of the potential helper. In the solo pulling control (day 7), no partner was present in the adjacent kennel. (**a**) Direct reciprocity. The focal dog (black) experienced help from a partner dog (A) that pulled (cooperator treatment), or no help from another partner dog (B) that did not pull (non-cooperator treatment). (**b**) Generalised reciprocity. Two different individuals provided help (C, D) or not (F, G,) where one partner (C, F) was present on the first day and the other partner (D, G) was present on the next day. On the test day, an unfamiliar dog (E, H) was presented. (**c**) Second experiment. The focal dog (black) either repeatedly experienced help from the same partner (I) and was subsequently tested in the role of the potential helper with this partner (direct reciprocity treatment), or it repeatedly received help from two different partner dogs (J, K) and was then tested with a new partner (L) (generalised reciprocity treatment). Both treatments were presented in random order.

When direct and generalised reciprocity were directly tested against each other with a new batch of dogs, the pulling frequencies did again not differ between the two paradigms (Wilcoxon test, n = 10, V = 24.5, p = 0.39; Fig. 1c), and neither did the latencies until the first pull, nor the median delays between pulls (Wilcoxon tests; latency: n = 10, V = 8, p = 0.181; median delay: n = 10, V = 23, p = 0.623; see electronic supplementary material, ESM, Fig. 1, Fig. 2). The pulling frequencies and median pulling delays of dogs applying direct and generalised reciprocity rules correlated highly with each other, suggesting that the dogs behaved similarly in both paradigms (Spearman rank correlations, both r_S_ = 0.991, n = 10, p < 0.01; ESM, Table 1, Table 2, Fig. 3, Fig. 4).

### Non-social control

In the solo pulling control, none of the 11 dogs of the first experiment pulled food towards the cage, and only 1 out of 10 dogs did so in the second experiment, which differed significantly from the cooperator treatment (Wilcoxon tests; first experiment: both, direct and generalised reciprocity: n = 11, V = 55, p = 0.005; second experiment, direct reciprocity: n = 10, V = 55, p = 0.006; generalised reciprocity: n = 10, V = 36, p = 0.014). The number of pulls in the first experiment did not differ between the non-cooperator and the solo pulling treatments (Wilcoxon tests, direct reciprocity: n = 11, V = 6, p = 0.181; generalised reciprocity: n = 11, V = 3, p = 0.346). The low propensity of dogs to pull in the solo pulling control suggests that pulling the rope was indeed a social service.

## Discussion

### Direct vs generalised reciprocity

Our data show that dogs reciprocate help, regardless whether their social partner is a known cooperator or an anonymous conspecific (Fig. 1a,b). In an iterated prisoner’s dilemma paradigm, dogs apparently apply the decision rule “help anyone if helped by someone”, which implies that they do not differentiate between direct and generalised reciprocity (Fig. 1c). In contrast, Norway rats show a higher cooperation propensity based on direct reciprocity than on generalised reciprocity^8^. There are several potential reasons for this difference between the two species, including their different social organisations, the domestication history of dogs, and different cognitive routines involved in dealing with social challenges. In any case, dogs seem to prefer using a simple rule of thumb when deciding about whether to help someone after having received help from a conspecific themselves. Such simple decision rule is also used by capuchin monkeys and humans^26^,^27^,^28^, species that, like dogs, clearly have the capability to use individual information about past experiences when deciding to provide or refuse help to a social partner. Even if the use of individual information may allow more prudent cooperation decisions, the costs of processing such information might outweigh the benefits, as predicted by statistical decision theory^35^,^41^,^42^.

Interestingly, our results suggest that familiarity is unimportant in decisions of dogs to return received help. At first glance, this seems to be at odds with results of a recent study of dog cooperation suggesting a positive effect of familiarity on the propensity to cooperate unconditionally, i.e. without previous cooperation experience^33^. However, this divergence makes sense when considering that unconditional cooperation can be evolutionarily stable among individuals that are either related with each other or significantly interdependent on each other, whereas reciprocity corresponds to an alternative cooperation mechanism that works independently of these conditions^18^,^37^,^43^,^44^. Familiarity is often used as a cue of relatedness^45^, which might explain why dogs pay attention to such cues when deciding to help a social partner unconditionally^33^. Another important difference between our study and the one of Quervel-Chaumette *et al*.^33^ is that we tested working dogs instead of family dogs. These working dogs were handled and trained by different people outside of the experiment. In contrast, family dogs are often handled mainly by one human partner, and frequently they do not easily associate with different people. Perhaps this different experience in interactions with humans might have facilitated the ability of working dogs to generalize also social experiences made with conspecifics, in comparison to family dogs. It is presently unknown, however, if this difference in the experimental subjects’ background influenced the results of these two studies.

### Non-social control

The results from our solo pulling control reveal that dogs do not simply copy the behaviour of a social partner with which they interacted during the experience phase. Like rats^46^,^47^, dogs seem to understand the need of a partner to make them act cooperatively (see also ref. 33).

### The significance of generalised reciprocity

Our results provide empirical evidence for the theoretical prediction that animals use simple “rule of thumb” solutions to solve complex problems^35^, as has been suggested also in other behavioural contexts^48^,^49^. As the application of generalised reciprocity requires only remembering and acting upon an individual’s own recent experience with an anonymous partner, the simplicity of this mechanism implies that it is can be applied quickly and with minimal cognitive costs, which makes it likely to be important in nature^8^,^18^,^20^,^29^. The few cases where generalised reciprocity has yet been tested in animals, including humans, have revealed that this mechanism is indeed applied when deciding about cooperative behaviour. As yet only in long-tailed macaques this could not be confirmed, but the statistical power of this study was very low (5 subjects tested^50^).

An important message conveyed by our results is that if animals are found to reciprocate help with a social partner, this does not mean that they apply the rather demanding decision rule characterizing direct reciprocity. Hitherto, direct reciprocity by excluding that test subjects use the simpler generalised reciprocity rule has been demonstrated only in humans^26^ and Norway rats^8^. In all other studies showing that animals reciprocate help to social partners, test subjects might actually have applied the simple rule “help anyone if helped by someone”, like our dogs did, because this possibility was actually not excluded by the performed experiments. Until this is properly tested, it seems more likely that animals assumed to show direct reciprocity in fact apply the simpler generalised reciprocity rules, because this mechanism is simple and cheap and was shown to establish evolutionarily stable cooperation in natural populations^19^,^20^,^21^,^22^,^23^,^24^,^25^.

## Materials and Methods

### Experimental subjects

All 41 dogs used in these experiments were unrelated, uncastrated males of the same breed (Belgian Shepherd, Malinois) and a limited age range (13–48 months) from the Swiss military service. These dogs represent a homogenous sample because of very similar housing conditions and use as working dogs. 11 focal dogs were chosen for the first experiment and 10 different focal dogs were used for the second experiment. The experimental procedure was authorized by the Swiss Federal Veterinary Office (license BE82-11) and therefore performed in accordance with relevant guidelines and regulations.

### Pre-experimental training

The experimental setup (Fig. 2a,b,c) was based on an IPD paradigm resembling that used for studies of reciprocity in Norway rats^8^,^29^,^51^. Two dogs were each placed in a kennel of 1.5 metres × 1.5 metres, at a distance of 60 centimetres between the two kennels to prevent physical contact. The pre-experimental training consisted of two phases.

#### Pulling food

The dogs learnt to pull a moveable platform towards themselves, without the presence of a partner, in order to receive a food item (one piece of dry food). This could be achieved by mouth-pulling a rope that was fixed to the platform. This phase lasted from one to six days, depending on individual abilities.

#### Pulling food for a partner

The dogs learnt to pull the platform for a social partner. In each trial, only one dog had access to the rope, thereby getting the opportunity to move the platform towards the kennel. Now, the pulling dog never got the reward himself, but only his partner. After successful pulling, the roles were immediately exchanged. The sequence of pulls was continuously increased during the training days until the dogs pulled 7 times in a row without being rewarded themselves in between. Described in more detail, a dog pulled once for his partner and then the roles were exchanged. If this worked, we gradually increased the number of pulls before exchanging the roles, first to two pulls before switching, then to three pulls, and so forth, up to seven pulls before switching the roles. Thereby the dogs learnt that a partner dog can help them to receive a reward and that they could be rewarded for their pulling after the roles were exchanged. The dogs from the first experiment needed 15 to 19 days (with 2 trainings per day) until they reached the criterion of seven pulls in a row. The dogs from the second experiment needed 14 to 15 days (again with 2 trainings per day).

#### Preparation of cooperative and non-cooperative partners

In addition to the 21 focal dogs, 20 dogs (12 in the first experiment and 8 in the second experiment) were randomly assigned to act as cooperators or non-cooperators (6 + 6 in the first experiment, 4 + 4 in the second experiment). Cooperators were trained exactly in the same way as the focal dogs. Before each experience phase the cooperator stooge was rewarded from the tray - before the focal dog was present - to ensure that they were motivated to pull for the partner. Non-cooperators were not taught how to pull food for a partner. Additionally, the platform was fixed to prevent an accidentally movement of the platform. But non-cooperators were similarly made familiar with the kennel and also got rewarded from the platform through a cooperator to ensure that they were motivated by the food item placed on the platform and hence behaved like cooperators, but without pulling food for the focal subject.

### Experimental setup

In all experimental trials, the focal dogs and their social partners were unfamiliar to each other. i.e. they had not met before. All experimental trials and the social control lasted for 5 minutes or until the dog had pulled 7 times, whichever occurred first. A pull was counted when the dog took the rope in the mouth and pulled the wooden board into the kennel. It was not necessary to conduct the experiment blindly regarding experimental predictions, because the pulling response was unmistakable. The experimenter was sitting behind a wall, so dogs could not obtain human cues when solving the task.

#### Direct and generalised reciprocity tested separately

The first experiment used a full-factorial design with each dog experiencing all treatments in a randomized sequence. During the experience phase, all focal dogs experienced either a cooperative or a non-cooperative partner that either provided food for them or not by pulling the movable platform into the focal dog’s compartment. Thereby, the focal dog either received food (from the cooperative partner) or not (from the non-cooperative partner). When the focal subject was paired with a non-cooperative partner, it received 7 pieces of food from the tray after the partner had been removed, to exclude that a potential difference in the response to cooperative and non-cooperative partners is merely based on food conditioning^46^. In the test phase, the roles were exchanged and focal dogs were allowed to pull for the partners they had previously experienced (direct reciprocity paradigm), or for unknown conspecifics (generalised reciprocity paradigm). To clarify whether dogs understand the social context of this experiment, for a non-social control on the last day of the experiment focal dogs could pull the platform when the neighbouring cage was empty, while still being unable to reach the reward by themselves (Fig. 2a,b; cf. 46).

#### Direct and generalised reciprocity tested against each other

In the second experiment, the two paradigms (direct and generalised reciprocity) were conducted intermittently with each focal dog in a random sequence, only with cooperative partners (Fig. 2c). The focal dogs either received food from the same cooperative partner (direct reciprocity) or from different cooperative partners (generalised reciprocity). In the test phase, the roles were again exchanged and focal dogs could pull for a previously cooperative partner (direct reciprocity) or for an unknown conspecific (generalised reciprocity). A non-social control was again performed on the last day.

### Statistical analyses

We used Wilcoxon matched-pairs signed-ranks tests to analyse the difference in pulling frequencies between the paired treatments (cooperative vs non-cooperative partner in the first experiment; same cooperative partner vs unknown partner in the second experiment). Additionally, we analysed the pulling frequencies of the first experiment in dependence of the experienced cooperation/defection and the type of reciprocity tested and their interaction with a generalized linear mixed model (GLMM), assuming poisson distributed data, that was corrected for overdispersion and included individual identity as random effect. The data were analysed with the statistics program R (R Development Core Team 2009; version 2.13.1).

## Additional Information

**How to cite this article**: Gfrerer, N. and Taborsky, M. Working dogs cooperate among one another by generalised reciprocity. *Sci. Rep.* **7**, 43867; doi: 10.1038/srep43867 (2017).

**Publisher's note:** Springer Nature remains neutral with regard to jurisdictional claims in published maps and institutional affiliations.

## Supplementary Material

Supplementary Information
