# Supplementary material for: Working dogs cooperate among one another by generalised reciprocity
Source: Sci Rep. 2017 Mar 6;7:43867. doi: 10.1038/srep43867 (PMC5338352; doi:10.1038/srep43867)
Supplement: Supplementary Information [file srep43867-s1.pdf]

Electronic supplementary material of **Working dogs cooperate among one another by generalised reciprocity**

Nastassja Rieder[1] & Michael Taborsky[1]

[1] Behavioural Ecology, Institute of Ecology and Evolution, University of Bern, Wohlenstrasse 50a, CH-3032 Hinterkappelen, Switzerland

Correspondence:

nastassja.rieder@iee.unibe.ch

Supplementary Methods

Subjects

The Swiss army dogs were housed individually in indoor and outdoor kennels. From 5am to 1pm they were trained for protection or rescue tasks. The dogs were fed three or four times a day and water was provided ad libitum.

Supplementary Results

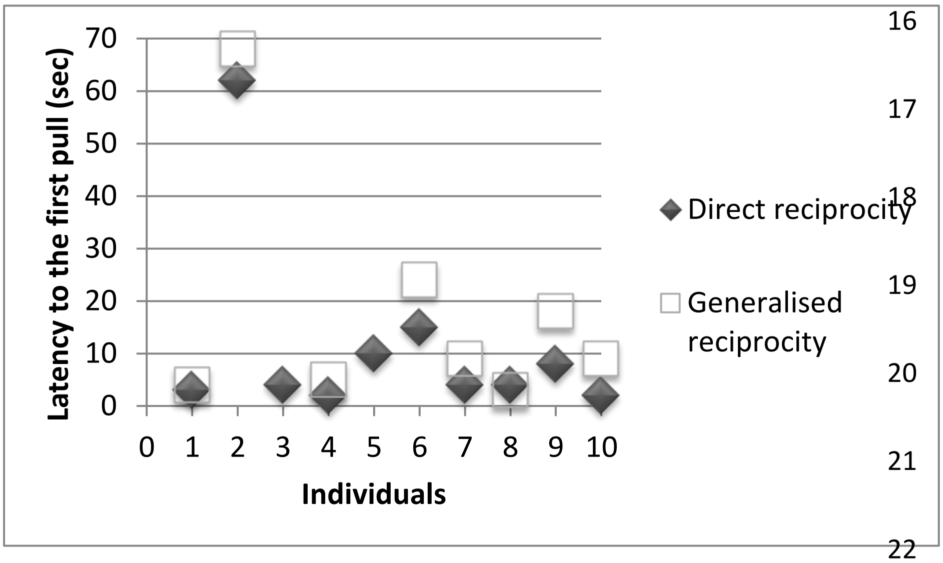

Figure ESM1. Latency (seconds) to the first pull in the second experiment. Two dogs (individuals 3 and 5) did not pull in the generalised reciprocity paradigm. There was no statistical difference in the latency to the first pull (Paired Wilcoxon signed-ranks test, n=10, V=8, p=0.181).

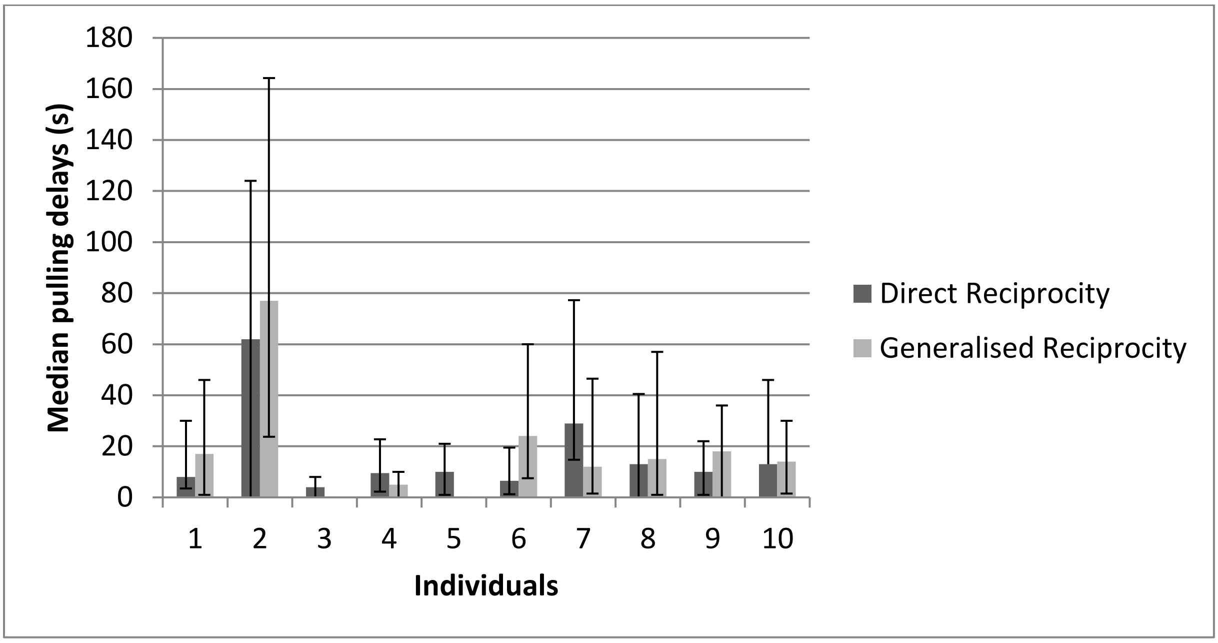

Figure ESM2. Median pulling delays (s) of the second experiment. There was no difference in the median pulling delays (s) between direct and generalised reciprocity (Wilcoxon, $p = 0.6228$).

DR	Rank	Binding	GR	Rank	Binding	Deviation	Deviation 2	
1	1	1.5	0	1	1.5	0	0	
1	2	1.5	0	2	1.5	0	0	
3	3	3	1	3	3	0	0	
4	4	4	4	4	4	0	0	
6	5	6	5	5	6	0	0	
6	6	6	5	6	6	0	0	
6	7	6	5	7	6	0	0	
7	8	9	6	8	8	1	1	
7	9	9	7	9	9.5	-0.5	0.25	
7	10	9	7	10	9.5	-0.5	0.25	
					Sum	0	1.5	rsp=0.991
								**p<0.01**

Table ESM1. Spearman's Rank-Order Correlation Coefficient of the second experiment.

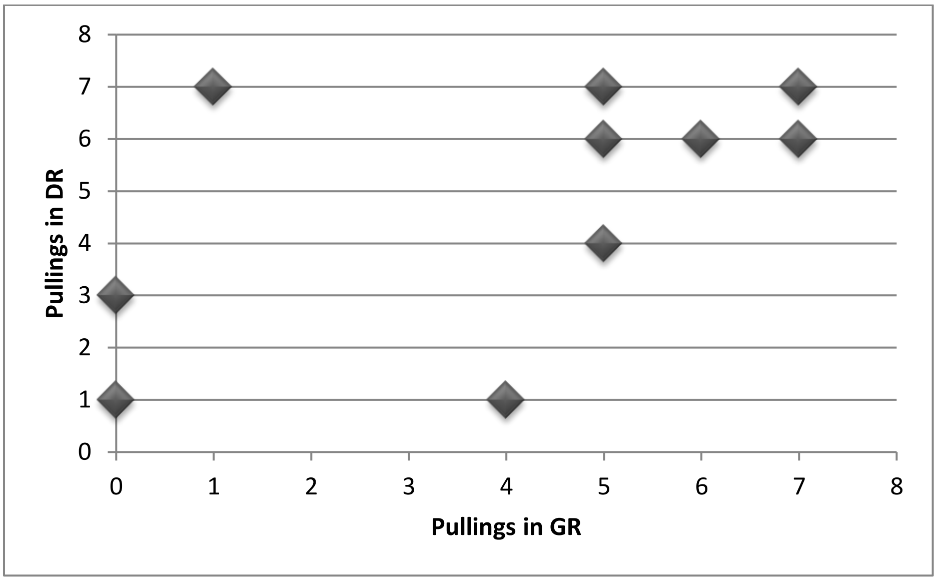

Figure ESM3. Relationship between pulling frequencies in the two test situations, direct (DR) and generalised (GR) reciprocity.

DR	Rank	Binding	GR	Rank	Binding	Deviation	Deviation 2	
4	1	1	0	1	1.5	-0.5	0.25	
6.5	2	2	0	2	1.5	0.5	0.25	
8	3	3	5	3	3	0	0	
9.5	4	4	12	4	4	0	0	
10	5	5.5	14	5	5	0.5	0.25	
10	6	5.5	15	6	6	-0.5	0.25	
13	7	7.5	17	7	7	0.5	0.25	
13	8	7.5	18	8	8	-0.5	0.25	
29	9	9	24	9	9	0	0	
62	10	10	77	10	10	0	0	
					Sum	0	1.5	
								rsp=0.991
								**p < 0.01**

Table ESM2. Spearman's Rank-Order Correlation Coefficient of median pulling delays (s).

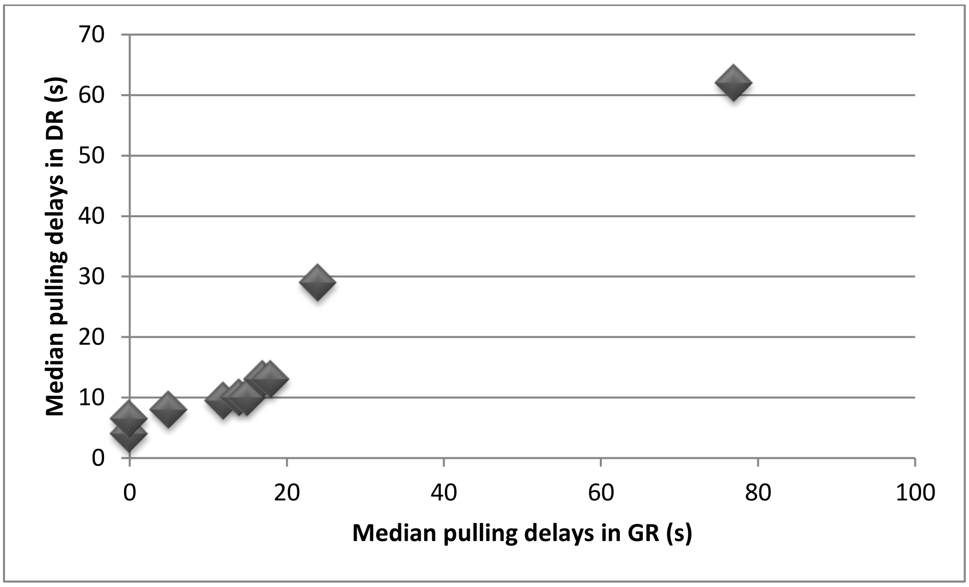

Figure ESM4. Relationship between median pulling delays (s) in the two test situations, direct (DR) and generalised (GR) reciprocity.

Supplementary Statistics

<u>Model and output of the GLMM analysis of the first experiment:</u>

R-code: glmer(Pulls~Cooperator*DR.GR+(1|Dog)+(1|Data_ID), family="poisson")

	Estimate	Std. Error	z value	p value
Intercept	-0.9730	0.5290	-1.839	0.06589
Cooperator experience	1.5203	0.5645	2.693	0.00708
Reciprocity type	0.2273	0.6479	0.351	0.72567
Coop. exper.x recipr. type	-0.2549	0.7747	-0.329	0.74216

Table ESM3. Fixed effects of the model.

R-code: drop1(model, test="Chisq")

	Df	AIC	LRT	p value
Test result	1	143.62	0.10691	0.7437

Table ESM4. Output of Log-likelyhood ratio test comparing previous model with model lacking interaction term.
